# Refining cardiac resynchronization therapy: a comprehensive review on the role of advanced multimodality imaging

**DOI:** 10.3389/fcvm.2024.1406899

**Published:** 2024-12-18

**Authors:** Flavia-Mihaela Stoiculescu, Diana-Ruxandra Hădăreanu, Călin-Dinu Hădăreanu, Ionuț Donoiu, Cristina Florescu

**Affiliations:** ^1^ Doctoral School, University of Medicine and Pharmacy of Craiova, Craiova, Romania; ^2^ Department of Cardiology, Clinical Emergency County Hospital of Craiova, Craiova, Romania; ^3^ Department of Cardiology, University of Medicine and Pharmacy of Craiova, Craiova, Romania; ^4^ Department of Cardiovascular Surgery, Clinical Emergency County Hospital of Craiova, Craiova, Romania; ^5^ Department of Cardiology, Filantropia Clinical Hospital of Craiova, Craiova, Romania

**Keywords:** cardiac resynchronization therapy (CRT), heart failure, multi-modality cardiac imaging, cardiac resynchronization therapy optimization, advanced echocardiography, cardiac magnetic resonance, cardiac dyssynchrony

## Abstract

Cardiac resynchronization therapy (CRT) offers significant benefits in symptom alleviation, reduction of rehospitalization rates, and overall survival of patients with heart failure (HF) with reduced ejection fraction (rEF). However, despite its proven efficacy, precisely identifying suitable CRT candidates remains a challenge, with a notable proportion of patients experiencing non-response. Accordingly, many attempts have been made to enhance patient selection, and to identify the best imaging parameters to predict the response and survival after CRT implantation. This review article provides a comprehensive overview on the role of multi-modality cardiac imaging in selecting, optimizing, and predicting CRT response and outcomes in HFrEF patients, beginning with an exploration of dyssynchrony types and their impact on HF progression, and an emphasis on the utility of echocardiography in assessing cardiac dyssynchrony. Subsequently, the role of advanced techniques such as speckle tracking and three-dimensional echocardiography, as well as the visual assessment of apical rocking (ApRock) and septal flash (SF) are highlighted. Finally, cardiac magnetic resonance (CMR) scar data, and novel modalities like four-dimensional flow CMR, together with single-photon emission computed tomography offer additional insights, emerging as valuable predictors of CRT response, and potentially refining the identification of suitable CRT candidates.

## 1 Introduction

Cardiac resynchronization therapy (CRT) is a cornerstone in the management of patients with heart failure with reduced ejection fraction (HFrEF), and numerous clinical trials have demonstrated the effects of CRT implantation in reducing HF symptoms, decreasing hospitalization rates, and improving patients’ survival (1). CRT implantation is recommended in patients with left ventricular (LV) ejection fraction (EF) ≤35% who remain symptomatic despite optimal medical therapy for at least 3 months, and who are in sinus rhythm with either a class IA in patients with a QRS duration ≥150 ms and left bundle branch (LBBB) morphology, or IIaB indication if the QRS duration is 130–149 ms or in case of a non-LBBB morphology (2). Furthermore, significant attempts were made in order to appropriately select the ideal candidates for CRT implantation, and to predict HFrEF patients’ outcomes after CRT implantation based on electrocardiographic parameters and different imaging techniques (mainly echocardiography). However, the number of CRT non-responders remains high, with a notable 30%–40% non-responder rate (3), and with a high variability of the response as well (4), and none of these measures increased the responder rate (5) or predict major adverse cardiovascular events (MACEs). Nevertheless, the suggested parameters were deemed insufficiently sensitive or specific (6) to decrease non-response, resulting in unnecessary pacing and elevated mortality rates in HFrEF patients. Consequently, the aim of this article is to provide a state-of-the-art review on the role of cardiac imaging for selecting, optimizing and predicting CRT response and outcome in HFrEF patients, from the basic principles of evaluating cardiac dyssynchrony, to the role of advanced multi-modality imaging.

## 2 Pathophysiology and types of dyssynchrony

The main determinant of the CRT response is the degree of LV dyssynchrony (7), playing a crucial role in the development and progression of HF due to its effects on the systolic and diastolic LV function, and right ventricular (RV) and left atrial (LA) function as well (8–10). Cardiac dyssynchrony encompasses both electrical and mechanical dyssynchrony. The electrical component is characterized by prolonged conduction time in the ventricles, manifesting as an increased QRS duration. In contrast, the mechanical one involves the discordant mechanical coordination, often marked by simultaneous contraction and stretching in various segments of the LV, with delays in the time to peak contraction from one segment to another. From another point of view, cardiac dyssynchrony types cand be classified in atrioventricular, interventricular, and intraventricular (11). The atrioventricular dyssynchrony impacts ventricular diastolic filling, because of the initiation of the ventricular contraction while still in the diastolic period, resulting in mitral regurgitation (12), a shortened ventricular filling time, and ultimately, atrial systole occurring during the early passive filling phase (13). The interventricular and intraventricular dyssynchrony have a greater impact on the ventricular pump function, and characterize the electro-mechanical alterations found in patients with LBBB, and their hemodynamic consequences manifest as a reduction in stroke volume, diminished stroke work, higher LV pressure, and an increase in LV end-systolic wall stress (14). The presence and degree of echocardiographic mechanical dyssynchrony before CRT proved to be a significant predictor of long-term survival, and patients who experienced resolution of mechanical dyssynchrony within the 12 months following CRT exhibited the most favorable outcome (15).

## 3 Definition, types and sex differences in CRT response

While CRT non-responders display some of the worst outcomes in the HF population, the concept of a CRT responder should be applied with caution. While response typically refers to an improvement in cardiac size or function and/or clinical improvement based on symptoms, the specific measures used assess it vary across studies, and there is no universal consensus on a clear definition to what CRT response is (16, 17). Several clinical endpoints such as New York Heart Association (NYHA) functional class, quality of life scores, and exercise capacity measured by the 6-minute walking distance, along with the hemodynamic response and echocardiographic increase in LVEF or reduction in LV size, and outcome measures assessment have been used to assess the effectiveness of CRT and define responders (18). However, while a 15% reduction in LV end-systolic volume (ESV) and an increase in LVEF of 5% are commonly accepted markers of CRT success (19), it may not uniformly apply across patients subgroups, because of the underlying myocardial damage and limited potential for reverse remodeling in ischaemic cardiomyopathies, in which it might represent a clinically significant improvement, in contrast to patients with a non-ischaemic etiology of HF where greater reversibility is often expected. This highlights the need for a more nuanced approach to evaluating CRT response that incorporated factors such as etiology of HF, sex differences, baseline ventricular function, and other clinical conditions, as a one-size-fits-all criterion may not be appropriate for every patient (20). Moreover, while certain conditions such as ischemic cardiomyopathy, atrial fibrillation, non-LBBB QRS morphology are linked to poorer clinical outcomes or less favorable LV reverse remodeling (20), the influence of sex on CRT response has only been hypothesized until recently. Cheng et al. performed a meta-analysis on 72 studies comprising 33,434 patients and found that women experienced greater reduction in the risk of all-cause mortality, cardiac death, HF hospitalization after CRT compared to men, along with consistently stronger echocardiographic evidence of reverse remodeling (21). The possible explanation of why women with LBBB QRS morphology, particularly if the QRS duration is 130–149 ms, show a significantly better response to CRT than men (22), is represented by the sex differences in LV size, as smaller LV size in women accounts for a lower QRS duration threshold in women for CRT benefit (23). Moreover, the presence of LBBB and non-ischemic cardiomyopathy is higher in women, while the prevalence of an ischemic etiology is higher in men, together with non-LBBB conduction abnormalities, a history of atrial tachyarrhythmias, several comorbidities such as chronic pulmonary obstructive disease (24), diabetes and renal dysfunction (25).

## 4 Electrocardiography

The use of 12-lead electrocardiograms (ECGs) has greatly enhanced the understanding of ventricular conduction abnormalities, and represents the basis of electrical dyssynchrony evaluation. Currently, the CRT implantation in HF class I and II indications according to clinical practice guidelines are established based on the QRS duration and morphology (2). However, one of the main limitations in selecting the CRT recipients based on the ECG is a large variability of LBBB definition depending on the criteria used (26). Moreover, the prevalence of LBBB morphology identification in the general CRT population differs widely—from 29% according to the American Heart Association/American College of Cardiology/Heart Rhythm Society (AHA/ACC/HRS) definitions (27), to 47% using European Society of Cardiology (ESC) criteria (28), and to 61% according to Strauss et al. (29). The robustness of the association between LBBB morphology of the QRS and outcomes after CRT varies based on the ECG classification used for defining it, with the simplest ones showing the strongest correlations with the clinical endpoints (30). Yet, even more notable is the absence of a significant correlation between QRS complex morphology and CRT outcomes as reported by a meta-analysis of 3,782 patients including five major randomized CRT trials (CARE-HF, RAFT, MIRACLE, MIRACLE-ICD, and LBBB REVERSE) (31). These findings contrast with the previously held belief that patients with LBBB were most favorable candidates for CRT, a view supported by the results of a large meta-analysis that included 6,523 patients from five trials (COMPANION, CARE-HF, MADIT-CRT, RAFT and REVERSE) which further emphasized that CRT did not improve the outcome of death and/or hospitalization for HF in non-LBBB morphology (32).

On the other hand, vectorcardiography offers a three-dimensional view of the heart's electrical vectors, providing additional information on the direction and magnitude of electrical forces. This can be particularly useful in cases where the standard ECG morphology is ambiguous or when assessing complex conduction disturbances (33). Another difficulty is represented by the non-LBBB patients since they cannot be treated as a single, homogenous group, other ECG parameters being particularly useful in this group of CRT candidates. The QRS area measured on the baseline 12-lead ECG was strongly associated with the clinical response and LV reverse-remodeling after CRT in both LBBB and non-LBBB patients with QRS ≥150 ms, and had better prognostic value compared to QRS morphology and duration (34). Finally, a reduction in QRS area after CRT is linked to lower mortality rates (35).

## 5 Echocardiography

Echocardiography remains the main imaging method used for selecting the patients that might benefit the most from CRT implantation, and for device optimization (36). However, the use of cardiac imaging to assess mechanical dyssynchrony is recommended only in patients with large QRS (>130 ms) as the Echo-CRT trial indicated potential harm when used as a criterion for CRT in patients with a QRS duration <130 ms (37). Table 1 provides a summary of the main echocardiographic parameters used for the evaluation of cardiac dyssynchrony, as well as their advantages, disadvantages and clinical usefulness.

**Table 1:** The main echocardiographic parameters used for the evaluation of cardiac dyssynchrony.

Index	Method	Advantages	Disadvantages	Prognostic value
SPWMD ≥130 ms	M-mode color TDI	• No need for advanced technical specifications • Easy to apply • Widely available	• Influenced by passive movements or wall tethering • Affected by akinetic segments	• Predictive of reverse remodeling and improvement in heart failure status
DFT/RR ≤40%	PW Doppler	• Marker of global myocardial performance	• Significantly influenced by heart rate	• Its increase after CRT reflects favorable reverse remodeling and is associated with better clinical outcomes
IVMD ≥40 ms	PW Doppler	• No need for advanced technical equipment or software • Widely available • High reproducibility	• Influenced by both LV and RV contraction and relaxation	• Good feasibility and reproducibility • Predicts survival and CRT response
Basal septal-to lateral wall delay ≥65 ms	Color DTI Ts	• Easy to apply • Analysis can be conducted offline	• Influenced by passive movements or wall tethering	• Highly predictive for both clinical and echocardiographic response after CRT
Maximum difference in Ts ≥100 ms	Color DTI Ts	• Analysis can be conducted offline • Enhanced identification of longitudinal dyssynchrony	• Influenced by passive movements or wall tethering	• Highly predictive for both clinical and echocardiographic response after CRT
Dyssynchrony index/Yu index ≥33 ms	Color DTI	• Analysis can be conducted offline • Enhanced identification of longitudinal dyssynchrony	• Influenced by passive movements or wall tethering	• Independently associated with long-term prognosis after CRT • Useful risk-stratification tool
SDI ≥8.3%	RT3DE	• Angle-independent assessment of regional and global deformation • Assesses dyssynchrony with a single aquisition	• Lower spatial resolution • Longer learning curve and the need for an experienced user • Requires offline analysis • Not widely available	• Conflicting data regarding its prognostic value

CRT, cardiac resynchronization therapy; DFT, diastolic filling time; IVMD, inter-ventricular mechanical delay; LV, left ventricle; PW, pulsed-wave; RR, cardiac cycle duration; RT3DE, real-time three-dimensional echocardiography; RV, right ventricle; SDI, systolic dyssynchrony index; SPWMD, septal to posterior wall delay; TDI, tissue doppler imaging; Ts, time to peak systolic velocities in the slowest of 6 basal LV segments.

### 5.1 M-mode, Doppler and two-dimensional echocardiography

LV intraventricular dyssynchrony can be evaluated by M-mode echocardiography using a simple index of septal to posterior wall motion delay (SPWMD), with a cut-off value of >130 ms identifying the patients with a more favorable outcome after CRT (38). The value of SPWMD was first demonstrated by Pitzalis et al. more than 20 years ago in a study on 20 patients, showing that a SPWMD of >130 ms and a QRS duration of >150 ms correlated with a positive response to CRT, defined as a >15% reduction in LV ESV index in 79% of the patients (39). A SPWMD of ≥130 ms furthermore predicted the improvement in LVEF and was associated with a lower risk of clinical worsening after CRT (40). This led to SPWMD emerging as a reliable prognosticator of LV reverse remodeling following CRT implantation. However, the feasibility and reproducibility of SPWMD measurements are limited according to a retrospective analysis of the CONTAK-CD trial which included 79 patients with HFrEF (EF 22 ± 7%, QRS duration 159 ± 27 ms). Furthermore, greater SPWMD values did not correlate neither with the six-month change in LV end-diastolic volume (EDV) and ESV index or LVEF, nor with any markers of clinical improvement, and no significant differences in SPWMD values were found between CRT responders and non-responders (41). Therefore, the utility of M-mode evaluation of LV dyssynchrony is supplemental to other echocardiographic modalities, and should not be used alone.

**Figure 1:**
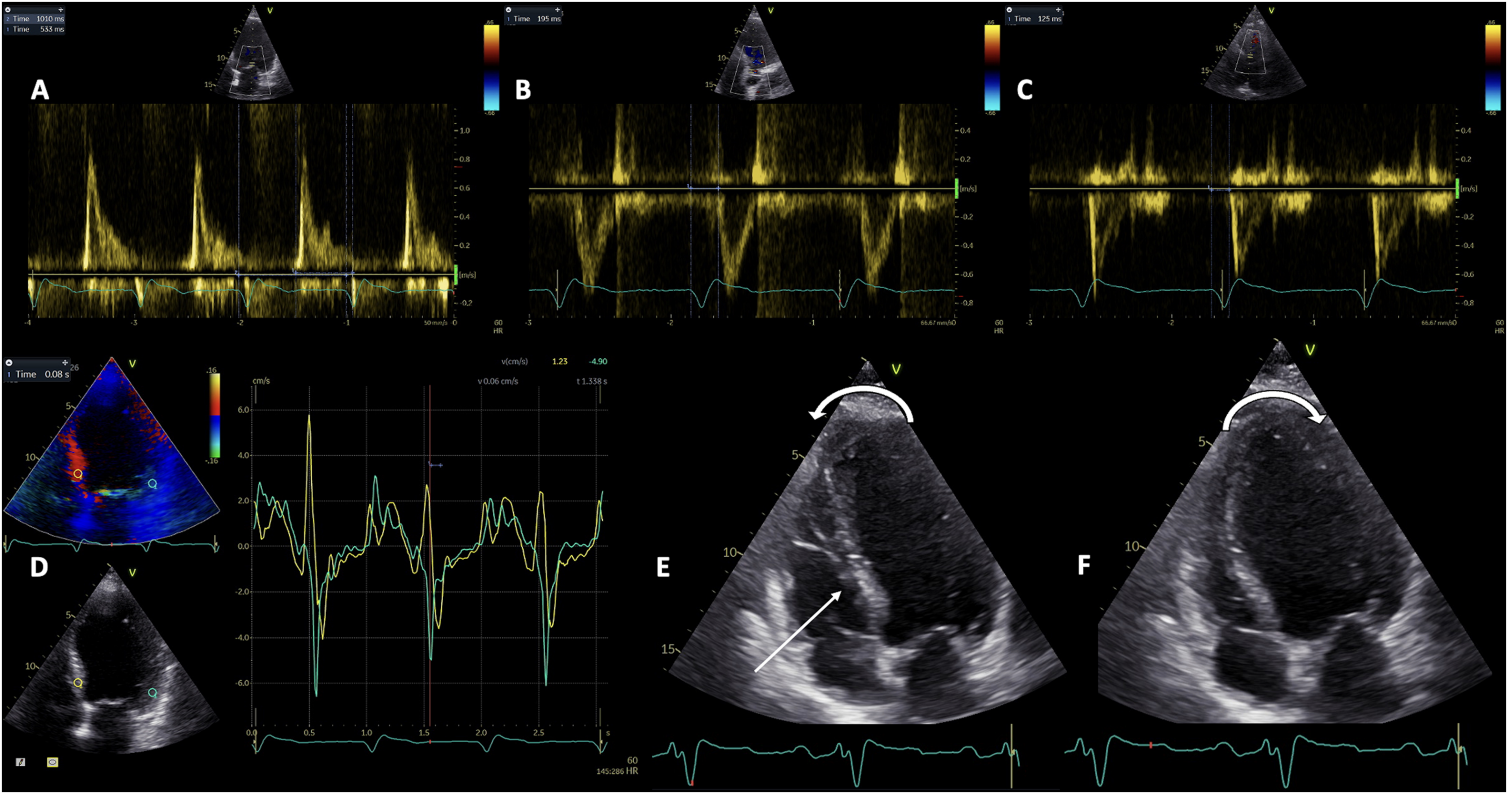
The evaluation of the three types of mechanical dyssynchrony by Doppler echocardiography. Pulsed-wave Doppler assessment of atrioventricular dyssynchrony as the diastolic filling time relative to cardiac cycle duration **(A)**; and interventricular dyssynchrony as the difference between LV pre-ejection time **(B)**, and RV pre-ejection time **(C)** Tissue Doppler Imaging evaluation of intraventricular dyssynchrony as the basal septal to lateral wall delay **(D)** Representation of septal flash and apical rocking **(E** and **F)**. LV, left ventricle; RV, right ventricle.

Pulsed-wave (PW) Doppler echocardiography is used for the assessment of all atrioventricular, inter- and intra-ventricular dyssynchronies. Atrioventricular (AV) dyssynchrony is objectivated by a reduced diastolic ventricular filling time, measured by PW Doppler at the level of the mitral valve leaflets’ tips between the onset of the E wave and the end of the A wave, and normalized as a percentage of the cardiac cycle (Figure 1A). A LV filling time <40% indicated significant AV dyssynchrony (42). It can be used only in sinus rhythm and results either from an abnormal delay between the end of atrial systole and onset of ventricular systole in case of a long PR interval, or from a prolonged and abnormal intraventricular conduction (43). Parsai et al. hypothesized that the identification of all types of dyssynchrony would better determine the CRT responders, and conducted a study on 161 patients investigated before and after CRT. They propose an algorithm that includes the identification of 4 subgroups of mechanisms: the presence of true dyssynchrony as SF, impaired diastolic filling with either short or long AV delay, and exaggerated LV-RV interaction. The CRT clinical response depended on correcting the underlying mechanisms involved in the development of HF, and solely relying on the assessment of LV dyssynchrony failed to identify 40% of responders (44).

Empirically, the interventricular dyssynchrony was considered the interventricular mechanical delay (IVMD), calculated as a difference between LV pre-ejection interval (Figure 1B), and RV pre-ejection interval (Figure 1C) of more than 40 ms. LV and RV pre-ejection intervals are measured by PW Doppler, from the onset of the QRS complex and, respectively, the initiation of aortic and pulmonary ejection flows (45). Several studies confirmed the association between the IVMD and a favorable response to CRT. In the SCART Study Achilli et al. found that an IVMD >44 ms independently predicted the response (46), while patients with IVMD >49 ms benefited significantly from CRT in an analysis by Richardson et al. of the CARE-HF trial (47). However, the IVMD is considered to lack sufficient accuracy to be used for CRT reponse in clinical practice according to the PROSPECT study (6). More recently, as part of the CAVIAR response score developed by the investigators of the MARC study, the vectorcardiographic QRS area, IVMD and ApRock were strongly associated to LV reverse remodeling after CRT (48). The MARC study, which is the only prospective multi-modality biomarker study on CRT response, provided significant insights into the effectiveness of various echocardiographic criteria used, underscoring the importance of combining different parameters to improve the accuracy of CRT response prediction.

Intraventricular dyssynchrony can be evaluated by measuring the prolongation of the LV pre-ejection interval, as well as that of the contraction of either the septum or the left lateral wall after aortic valve closure (49).

Similarly, RV dyssynchrony indices in CRT patients were retrospectively evaluated. Adding the measurement of RV indices provides incremental prognostic value compared to LV parameters, with the highest sensitivity and specificity for RV deformation synchrony and RV isovolumic contraction dyssynchrony. However, they did not predict reverse remodeling after CRT (50). In contrast, the non-invasive estimation of RV to pulmonary artery coupling measured as the ratio between tricuspid annulus systolic excursion and systolic pulmonary artery pressure (TAPSE/sPAP) predicted both the response to CRT and LV reverse remodelling, andCRT responders also had improved TAPSE/sPAP at follow-up (51, 52). Moreover, in a large study on 807 CRT recipients followed-up for a median time of 8 years, the rates of survival at 3 and 5 years were significantly lower in patients with a TAPSE/sPAP <0.45 mm/mmHg, showing poorer long-term outcomes in CRT patients with RV to pulmonary artery uncoupling (53).

While the impact of CRT on the diastolic function remains a topic of debate (54), certain prospective studies have indicated that a favorable filling pattern and a less enlarged LA at baseline are more likely to correlate with positive LV remodeling following CRT (55). Moreover, a lack of reduction in mean LV filling pressures after CRT was associated with a negative response (56). Grade I LV diastolic dysfunction lead to a better prognosis compared to grade II or III (54). Nonetheless, a smaller LA volume index per one unit of standard deviation below the mean predicted LVEF super-response after CRT (57).

Tissue Doppler Imaging (TDI) is essential for the accurate determination of the amplitude, timing of onset, and peak systolic and diastolic velocities in correlation with the ECG signal. PW TDI is useful for measuring the electromechanical delay (58, 59), and the electro-systolic delay (60), from the beginning of the QRS interval to S wave onset, and to peak systolic contraction, respectively. Additionally, color TDI loops can be recorded and subsequently analyzed offline as reconstructed signals, in order to overcome the well-known limitations of PW TDI. The color-coded TDI has been the method of choice for assessing dyssynchrony by echocardiography for many years (61). The dyssynchrony indices obtained by color TDI are basal septal to lateral wall delay (Figure 1D), maximum time to peak systolic velocity in the slowest of 6 basal LV segments, as well as the Yu index which integrates data from the 3 apical LV views and represents a 12-segment model (62). While Bax et al. elegantly demonstrated that the degree of LV dyssynchrony predicted the clinical response and LV remodeling after CRT, with a cut-off value of 65 ms for the opposite wall delay (63), a Yu index or mechanical dyssynchrony index ≥33 ms managed to predict LV remodeling in patients with a QRS duration >150 ms with a sensitivity of 100% and specificity of 78% (64). An alternative approach to the Yu index is calculating the time to peak systolic velocity in all the segments, for which a value ≥100 ms is predictive of the CRT response (61). However, the 12-segment model has higher variability and the disadvantage of being more technically challenging (65).

Myocardial strain imaging derived by TDI offers widely-used diagnostic tools, potentially enhancing patient selection for CRT. Currently, several strain parameters are used as clinical indicators of CRT response, the most frequently used parameter being the delayed longitudinal contraction or post-systolic shortening, defined as more than 30% of 12 LV segments contracting after aortic valve closure (62). Also, recent data showed that end-systolic septal strain strongly correlates with favorable reverse remodeling following CRT, regardless of the assessment technique employed. Utilizing any strain imaging technique to measure end-systolic septal strain offers additional predictive value beyond existing guideline criteria (66), yet TD strain imaging poses the well-known limitations of Doppler angle dependency and technical difficulties in patients with spherical LV geometry. Nonetheless, Yu et al. identified time to peak myocardial contraction as the strongest predictor of LV reverse remodeling (67) Finally, TD strain-imaging derived mechanical dispersion refers to the variation in the timing of myocardial contraction across the different LV segments. It is calculated as the standard deviation of time-to-peak contraction of these segments, with a higher mechanical dispersion index indicating more dyssynchrony. While newer techniques (i.e., speckle-tracking echocardiography) are increasingly used for assessing mechanical dispersion due to their higher spatial resolution and ability to provide more detailed, angle-independent measurements, the TDI-derived mechanical dispersion index remains a reliable and widely accessible parameter, and a strong predictor of outcomes in CRT recipients (67).

Echocardiographic and Doppler imaging methods remain the foundation of mechanical dyssynchrony assessment as a key factor for determining CRT eligibility, even though the results of the PROSPECT study taught that the complexity of the technical issues impact their feasibility and reproducibility, and no ideal method exists (65). However, since PROSPECT, several advancements in cardiac imaging (speckle-tracking echocardiography, three-dimensional echocardiography, cardiac magnetic resonance) have improved the accuracy and reliability of assessing dyssynchrony and overall cardiac function, reinforcing the expanding role of cardiac imaging in CRT.

### 5.2 Visual assessment of apical rocking and septal flash

Both ApRock and SF occur as a consequence of the mechanical dyssynchrony secondary to the LBBB (15), and their superiority over conventional parameters has already been demonstrated in several prospective observational studies (68, 69). ApRock (Figures 1E,F) is characterized as an initial septal contraction in the LV isovolumic contraction period which results in a short inward motion of the septum and causes the apex to move septally. Next, the delay in the activation of the lateral wall pulls the apex laterally during the ejection time while stretching the septum (68, 70). SF is caused by an initial thickening/thinning of the septum during isovolumic systole (Figure 1E). This phenomenon can also be easily identified using M-mode echocardiography in the parasternal long-axis view or tissue Doppler imaging in both the short and long parasternal long-axis views (71), while a low-dose dobutamine administration may help unmasking the SF in a minority of challenging cases (72).

The visual assessment of ApRock and SF is relatively easy and reproducible, and they should be used frequently in selecting CRT candidates because of their prognostic value. However, while their presence is associated with a favorable outcome in patients who undergo an upgrade from regular pacing to CRT, as well in patients with a QRS duration of less than 150 milliseconds (42), also having additional value in predicting long term major cardiac events (73), the accurate recognition of SF and ApRock in candidates for CRT is heavily dependent on the expertise of the echocardiographer (74).

### 5.3 Speckle tracking echocardiography (STE) and LV myocardial work

**Figure 2:**
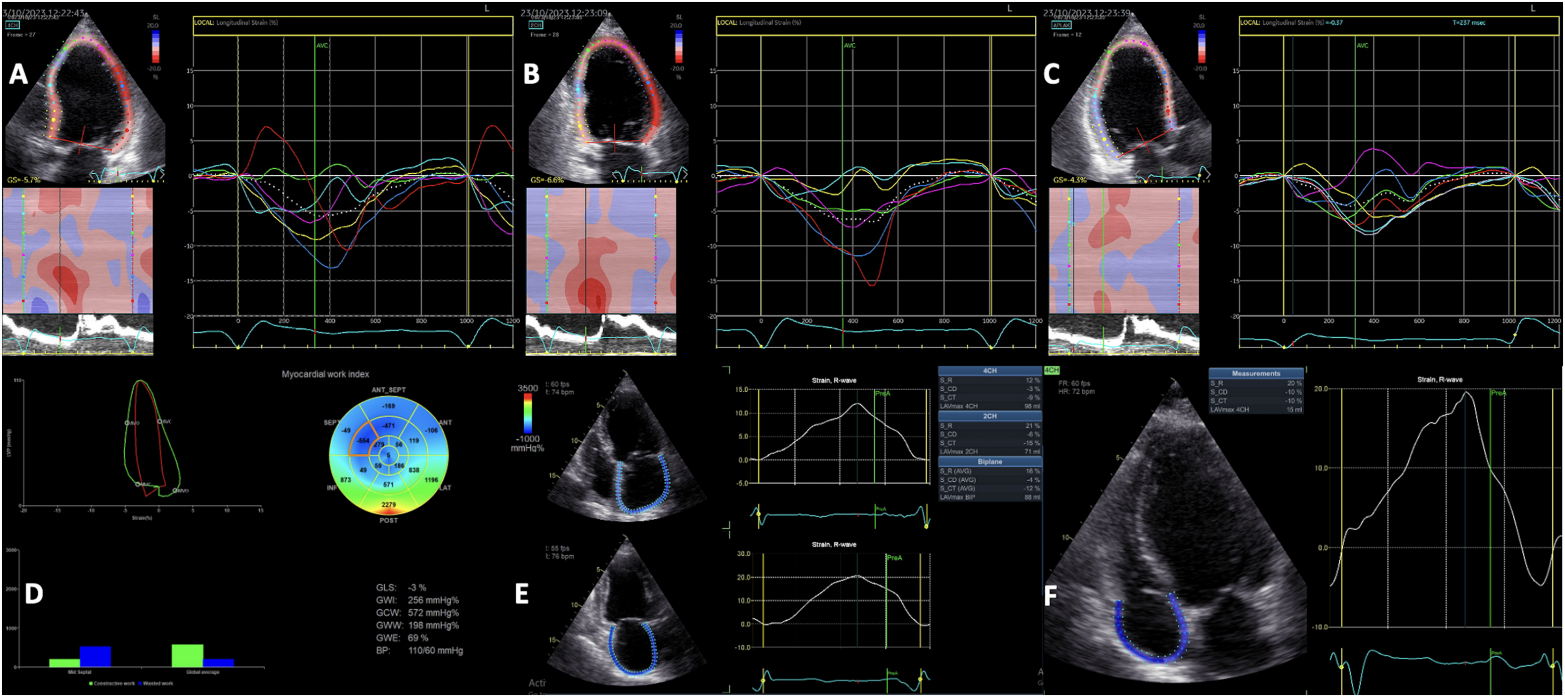
Two-dimensional speckle-tracking analysis of segmental left ventricular myocardial deformation showing the intraventricular dyssynchrony in the apical 4-chamber **(A)**, 2-chamber **(B)** and 3-chamber **(C)** views. Myocardial work analysis showing increased GWW, and regional WW at the level of the septum, and low GWI, GCW and GWE **(D)** Left atrial strain analysis **(E)** and Right atrial strain analysis **(F)** showing reduced values in a patient with left ventricular dyssynchrony and left bundle branch block. GCW, global constructive work; GWE, global work efficiency; GWI, global work index.

Mechanical dyssynchrony, rather than electrical dyssynchrony, serves as the primary predictor of responsiveness to CRT. On one hand, electrical dyssynchrony, evaluated by QRS duration on an ECG, may be less reliable due to scar-related moderate QRS enlargements that may not correspond to significant mechanical dyssynchrony. This phenomenon is especially noticeable among patients with an ischemic etiology of HF, in which several myocardial segments have delayed contraction, often attributed to scar tissue formation. On the other hand, scar tissue or fibrosis, resulting in reduced or lack of contractile reserve, influence CRT response. However, relying solely on time-delay indexes for identifying responders is inherently limited since it does not consider residual myocardial contraction. Accordingly, comprehensive echocardiographic evaluations of both LV mechanical dyssynchrony (Figures 2A–C) and contractile function, providing insights into myocardial viability and scar tissue burden, can now be conducted reliably and independently of imaging angles through the application of STE (75, 76). Delgado et al. proved that combining the LV radial dyssynchrony with the radial strain of the LV segment corresponding to the LV lead placement (with values <16.5% indicating a myocardial scar with >50% transmurality as validated by cardiac magnetic resonance), and placing the LV lead in the latest activated segment defined as concordant lead position predicted the long-term survival in a large cohort of ischemic HF patients (77). These three parameters provided additional prognostic value beyond that offered by clinical parameters alone. However, radial dyssynchrony cannot be used in patients with a history of septal infarction.

LV global longitudinal strain (GLS) is a more reliable indicator of LV systolic performance compared to LV EF, simultaneously predicting cardiac events in CRT recipients (75). Greater baseline LV GLS values, and significantly higher LV GLS values at follow-up were found in CRT responders compared to non-responders (76). Moreover, 2D STE can identify the significant activation delay typical of a true LBBB, patients without the typical LBBB contraction pattern facing a threefold increase in the risk of adverse outcomes following CRT implantation (78). Another important index derived from STE is LV mechanical dispersion. Van der Bijl et al. demonstrated that greater LV mechanical dispersion at 6 months post-CRT predicted all-cause mortality and higher arrhythmic risk, independent of the clinical response and LV reverse remodeling, while baseline dispersion did not impact the outcome (79). However, LV mechanical dispersion does not differentiate between an ischaemic and conduction disturbance substrate, since it is common to observe the reduced systolic shortening and post-systolic shortening in the scarred myocardium (80, 81), and accordingly, mechanical dispersion is not currently recommended to be used for dyssynchrony assessments (42).

Adding LA reservoir strain (Figure 2E) measurement to LV GLS calculation is an useful tool for selecting CRT candidates, and could potentially improve the risk stratification in patients undergoing CRT implantation. Furthermore, higher LA reservoir strain at baseline correlates with a more significant LV remodeling after CRT (82, 83). Nevertheless, although frequently overlooked, right atrial (RA) remodeling, assessed as either RA volume or RA strain (Figure 2F), has important prognostic value in HF patients, including in those undergoing CRT (84, 85).

Strain delay index derived from longitudinal strain amplitude measurements by two-dimensional STE is another reliable predictor of CRT response, regardless of whether patients have an ischemic or nonischemic etiology of HF (86).

Moreover, the non-invasive echocardiographic LV myocardial work (Figure 2D) evaluation prior to CRT implantation has emerged as a valuable technique for the identification of CRT responders (87). Global wasted work and the average wasted work measured at the level of the interventricular septum derived from the echocardiographic LV pressure-volume loops had higher values in CRT responders compared to non-responders, and a significant reduction was observed after CRT implantation, converging towards the values typical of a normal heart (88).

The prognostic value of septal wasted work for the response to CRT may be enhanced by combining it with the LV wall motion score. The LV lateral wall to septal work difference alone had predictive value comparable to visual assessments of dyssynchrony, and combining it with septal scar evaluation by CMR significantly enhanced the accuracy of predicting CRT response (89). Finally, an effective parameter for the prediction of long-term reverse remodeling involves examining the redistribution of myocardial work between the septal and lateral LV walls following CRT implantation (90).

While the existing evidence on these echocardiographic parameters may not be robust enough to solely guide treatment decisions, routine analysis of myocardial work parameters should be integrated into the patient selection process because of their demonstrated value in selecting patients that might benefit from CRT, and an integrative approach might enhance the selection of suitable CRT candidates (91).

### 5.4 Three-dimensional (3D) echocardiography

**Figure 3:**
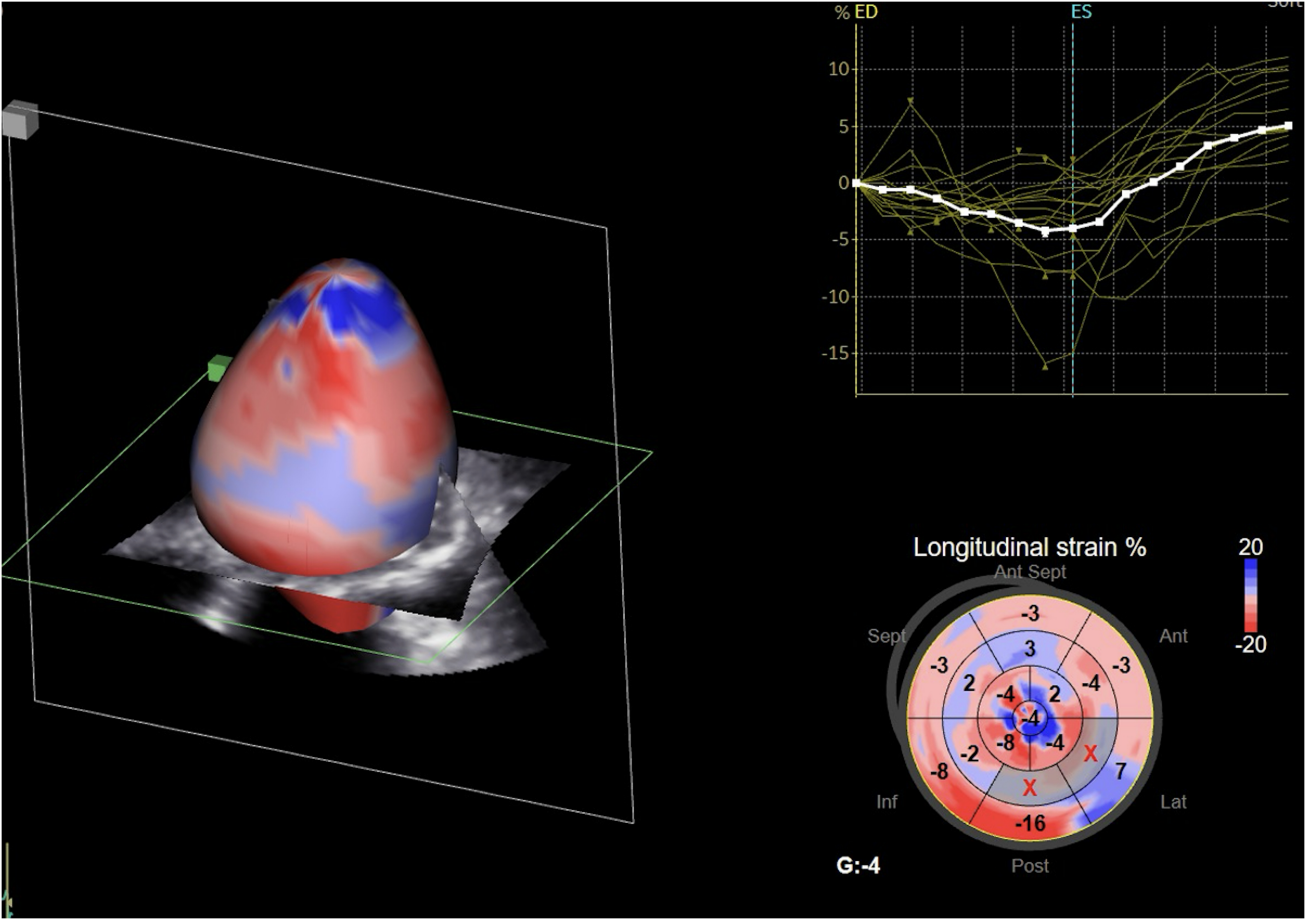
Three-dimensional speckle-tracking analysis of left ventricular global longitudinal strain.

Due to the intricate spatial orientation of LV myocardial fibers, and its simultaneous contraction in various directions, LV mechanics are recognized as a 3D phenomenon, and 3D echocardiography provides its most accurate evaluation (92–94). Despite the less encouraging outcomes reported in the PROSPECT trial (6), there is a growing interest in employing advanced echocardiography to identify patients who would benefit from CRT (93). The main additional value of 3D echocardiography is that it enables simultaneous comparison of synchrony across LV segments within the same cardiac cycle. An essential parameter derived from 3D echocardiography is the systolic dyssynchrony index (SDI) (95). The SDI represents the standard deviation of the average time intervals necessary for every LV segment to reach their minimum end-systolic volume. Expressed as a percentage of the entire cardiac cycle, this index is useful for comparing patients with different heart rates. Those with normal cardiac function demonstrate well-synchronized segmental function. Importantly, individuals who respond positively to CRT exhibit a significant reduction in SDI, corresponding to decreases in LV end-diastolic volume and increases in EF (96). Apart from SDI, various metrics obtained from 3D speckle-tracking echocardiography — namely, longitudinal strain (Figure 3), radial strain, circumferential strain, and, more recently, area strain — have been proposed for assessing myocardial mechanical dyssynchrony (97).

## 6 Cardiac magnetic resonance (CMR)

The utilization of cardiovascular magnetic resonance (CMR) imaging for HF evaluation is being used increasingly. This trend reflects the numerous appealing attributes of CMR in contrast to echocardiography, including enhanced tissue characterization, superior spatial resolution, and the absence of imaging limitations related to patient orientation and overlapping structures.

### 6.1 Tissue scar, myocardial viability and myocardial dyssynchrony evaluation

**Figure 4:**
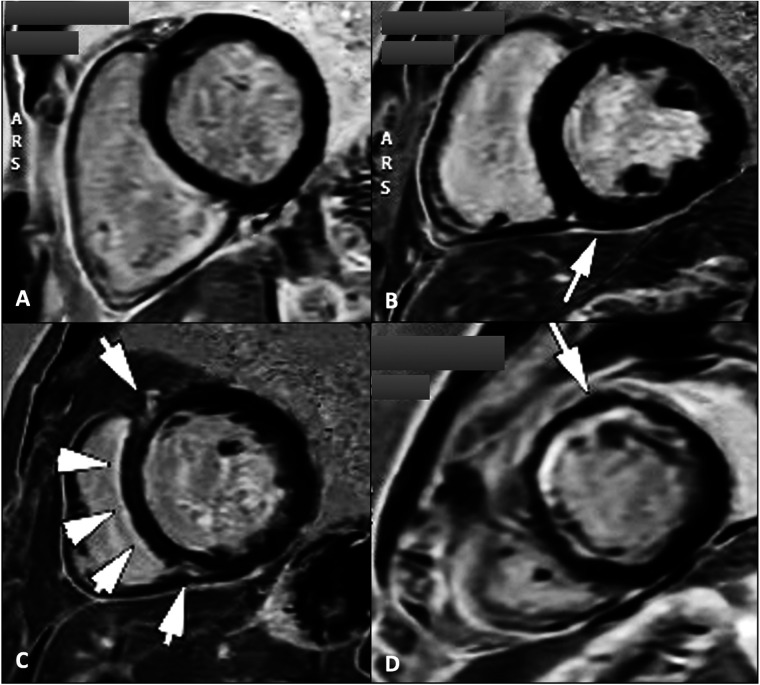
Cardiac magnetic resonance late gadolinium enhancement sequences for the assessment of myocardial scar tissue/fibrosis in four patients with dilated cardiomyopathy showing absence of LGE **(A)**; localized LGE at the level of the inferior intraventricular septum on the RV insertion point **(B)**; midwall linear fibrosis at the level of the intraventricular septum commonly known as “septal stripe” LGE, and at the level of the RV insertion points **(C)**; and subendocardial antero-septal and anterior wall LGE **(D)** types B and C LGE pattern are typically found in patients with genetic/idiopathic etiologies of dilated cardiomyopathies, while pattern D suggests an ischaemic etiology. LGE, late gadolinium enhancement; RV, right ventricle.

Evaluating the magnitude and location of the myocardial scar tissue by the use of late gadolinium enhancement (LGE) plays a crucial role (Figure 4), together with the evaluation of different LV contraction patterns (3). The identification and characterization of scar patterns is particularly valuable in predicting the clinical response in LBB pacing for CRT (98).

In patients with nonischemic dilated cardiomyopathy, significant myocardial fibrosis and reduced circumferential dyssynchrony at CMR were independently associated with unfavorable response and long-term events after CRT (99). On the other hand, in patients with ischemic cardiomyopathy, the size and position of the myocardial scar tissue significantly affect the response to CRT in two primary ways. Firstly, placing the LV pacing lead in scarred regions correlates with less clinical and echocardiographic improvements. Secondly, greater scar burden corresponds to reduced residual LV contractility (74). A substantial scar extent (>33%) or high transmurality (>51%) serve as unfavorable predictors for CRT response. Interestingly, lateral wall scarring was less predictive of CRT response than septal scarring. The presence of septal LGE, whether ischemic or non-ischemic, is a robust indicator for predicting non-response and unfavorable long-term outcomes after CRT (77, 100).

Furthermore, because mechanical dyssynchrony parameters are affected by regional scarring, several CMR methods have been developed for an adequate evaluation of dyssynchrony. Endocardial contour tracking software, contraction propagation maps, CMR-tissue synchronization indicesare some of the used sequences and analysis techniques.

This can be explained by the reduction in the typical LBBB -induced septal motion pattern (101) due to the decreased contractility of the lateral wall. The septum is thereby less stretched and SF and ApRock are diminished (102). Recent studies suggest that septal scar evaluation by CMR LGE together with SF visual assessment by CMR cine sequences as a singular imaging modality, together with other parameters such as delayed aortic valve opening measured relative to both end-diastole, and to pulmonic valve opening, or changes in septal-to-LV free-wall curvature ratios provide further insights into mechanical dyssynchrony, and accurately identify responders to CRT, while also reliably predicting long-term survival (103). If septal LGE is absent, the response rate remains outstanding, regardless of the presence or absence of other parameters of dyssynchrony. Conversely, if septal LGE is present without the occurrence of SF, the likelihood of a favorable response to CRT is significantly diminished, and if both septal LGE and SF are present, patients could respond positively to CRT (69).

Finally, integrating scar data with non-contact endocardial mapping to identify regions of slow conduction facilitates the optimization of the hemodynamic response. Furthermore, the combination of scar data with regional contractility patterns proves to be more effective in predicting long-term remodeling after CRT compared to standard echocardiography (104).

Accordingly, CMR evaluation plays a crucial role for selecting CRT candidates, predicting cardiac remodeling and patients’ outcomes after CRT implantation, additionally identifying the regions that should be avoided during the lead placement process. CMR is useful in establishing the indication for adding an implantable cardiac defibrillator (ICD) to CRT (CRT-D) for primary sudden cardiac death prevention (105), which might be particularly important as demonstrated by the findings from the MADIT-CRT study (106). In patients with mildly symptomatic HFrEF [classified as New York Heart Association (NYHA) class I or II if ischemic, and NYHA class II if non-ischemic], with an LVEF of 30% or lower, and a QRS duration of 130 ms the prophylactic treatment with CRT-D significantly lowers the risk (including mortality) in comparison to solely receiving an ICD (107). Lastly, the importance of using CMR together with cardiac computed tomography for creating detailed anatomical maps together with a thorough understanding of the ventricular anatomy, including the identification of structural abnormalities as part of the pre-procedural planning are crucial for precise lead placement in conduction system pacing (108).

### 6.2 Other CMR-derived measurements

Apart from myocardial scar and viability evaluation by CMR, greater CMR-derived circumferential uniformity ratio estimate used to quantify LV mechanical dyssynchrony by measuring LV segments contraction and stretch as negative, positive circumferential strain, respectively (109), was associated with a more favorable response and survival in female HF CRT patients (110). Nonetheless, both LA size and function (reservoir and booster function) (111), as well as RV function (RVEF, with a cut-off value of 55%) (112) predicted CRT response and LV reverse remodeling.

### 6.3 Four-dimensional (4D) flow CMR

Hemodynamic force (HDF) analysis of LV blood flow is a novel indicator of cardiac function that offers distinctive insights into the relationship between the ventricular movement and the resulting blood flow patterns, identifying HF patients with LBBB who are unlikely to benefit from CRT. Although not commonly used in clinical practice, it offers significant advantages in the comprehensive hemodynamic evaluation since it allows the assessment of complex flow dynamics in all three spatial dimensions over time. LV HDF represent the collective forces exchanged between the blood pool and the surrounding myocardium, which arise from the cumulative pressure gradients within the LV (113). In healthy hearts, LV HDF primarily align in the longitudinal direction, and an elevated ratio of transverse to longitudinal HDF suggests an aberrant blood flow pattern (114). CRT responders have higher inferior-anterior systolic and apex-base diastolic HDF (115).

The short-axis to long-axis 4D filling HDF ratio, an indicator of the deviation of the LV hemodynamic forces from the main flow direction, was higher during the initial diastolic filling phase in patients with dyssynchronous LV relaxation (LBBB patients) compared to age, gender, heart rate, and LV characteristics matched non-LBBB patients (116). However, LV HDF are influenced by conditions that lead to changes in LV inflow directions such as mitral valve dysfunction or prosthetic valve replacement, as well as significant regional wall motion abnormalities resulting from myocardial infarction. Consequently, HDF analysis may offer supplementary insights for the personalized evaluation of patients suitable for CRT.

## 7 Single-photon emission computed tomography (SPECT)

In cases when CMR is unavailable or contraindicated, SPECT may be used for myocardial scar tissue and viability evaluation in CRT recipients, and their identification has been linked to both CRT response and prognosis. Perfusion defects in the septal and apical segments that arise from relative hypoperfusion in the septal region compared to the lateral wall in the presence of LBBB, and in the absence of coronary artery lesions can also be detected by SPECT (117, 118).

## 8 Conclusions

A multiparametric evaluation is key for the personalized evaluation of HF patients undergoing CRT, and integrating multimodality cardiac imaging techniques has the potential to improve outcomes and reduce the number of non-responders. Echocardiography remains essential in evaluating cardiac dyssynchrony, with advanced techniques like speckle-tracking echocardiography and three-dimensional echocardiography improving patient selection. Cardiac magnetic resonance imaging provides complementary information on myocardial scar tissue, aiding in predicting CRT response and guiding lead placement. Finally, emerging modalities such as four-dimensional flow CMR offer novel perspectives on LV hemodynamic forces and LV blood flow patterns, potentially further refining the identification of suitable CRT candidates.
